# Multidimensional vulnerability among older adults in Germany: Social support buffers the negative association with life satisfaction

**DOI:** 10.1007/s00391-022-02142-3

**Published:** 2022-12-02

**Authors:** Volker Cihlar, Frank Micheel, Andreas Mergenthaler

**Affiliations:** Federal Institute for Population Research (BiB), Friedrich-Ebert-Allee 4, 65185 Wiesbaden, Germany

**Keywords:** Cumulative risk factors, Life satisfaction, Social support, Confirmatory factor analysis, GEDA 2014/2015-EHIS, Kumulative Risikofaktoren, Soziale Unterstützung, Konfirmatorische Faktorenanalyse, GEDA 2014/2015-EHIS

## Abstract

**Background:**

Multidimensional vulnerability among older adults, characterized by low levels of individual resources in different life domains, has been insufficiently studied. This phenomenon is considered to be associated with a marked decrease in overall life satisfaction. Social support is supposed to buffer the negative effect of multidimensional vulnerability on life satisfaction.

**Methods:**

Analyses are based on the German Health Update dataset (GEDA 2014/2015-EHIS). The analytic sample includes respondents ≥ 65 years (*N* = 5826). Confirmatory factor analyses were performed to construct a latent variable from the indicators income poverty, activities of daily living (ADL) limitations, multimorbidity, mental problems, and living alone. Multivariate linear regression models estimate the relationship between vulnerability and life satisfaction with a special focus on the interaction between vulnerability and social support.

**Results:**

The analyses supports the multidimensional construct of vulnerability. Social support considerably moderates the negative relationship between vulnerability and life satisfaction. As the degree of vulnerability increases, the influence of social support becomes more pronounced.

**Conclusion:**

The assessment of vulnerability as a multidimensional construct helps to depict the life situation of older people in a more differentiated way. Vulnerable older adults with a small or unreliable social network while finding it difficult to access practical help need additional external social support to achieve a high level of life satisfaction.

**Supplementary Information:**

The online version of this article (10.1007/s00391-022-02142-3) contains supplementary material, which is available to authorized users.

## Abstract (de)

**Hintergrund:**

Multidimensionale Vulnerabilität älterer Erwachsener, die durch ein geringes Maß an individuellen Ressourcen in verschiedenen Lebensbereichen gekennzeichnet ist, wurde bislang unzureichend untersucht. Dieses Phänomen kann mit einem deutlichen Rückgang der allgemeinen Lebenszufriedenheit einhergehen. Es wird vermutet, dass soziale Unterstützung die negativen Auswirkungen multidimensionaler Vulnerabilität auf die Lebenszufriedenheit abpuffert.

**Methoden:**

Die Analysen basieren auf dem Datensatz „Gesundheit in Deutschland aktuell“ (GEDA 2014/2015-EHIS). Die analytische Stichprobe umfasst Befragte ≥ 65 Jahre (*n* = 5826). Mit Hilfe von konfirmatorischen Faktorenanalysen wird eine latente Variable aus den Indikatoren Einkommensarmut, Einschränkungen in den Aktivitäten des täglichen Lebens (ADL), Multimorbidität, mentale Probleme und Alleinleben konstruiert. Multivariate lineare Regressionsmodelle schätzen den Zusammenhang zwischen Vulnerabilität und Lebenszufriedenheit mit besonderem Augenmerk auf die Interaktion zwischen Vulnerabilität und sozialer Unterstützung.

**Ergebnisse:**

Die Analyse bestätigt das multidimensionale Konstrukt der Vulnerabilität. Soziale Unterstützung moderiert die negative Beziehung zwischen Vulnerabilität und Lebenszufriedenheit erheblich. Mit zunehmendem Grad der Vulnerabilität wird der Einfluss der sozialen Unterstützung deutlicher.

**Diskussion:**

Die Betrachtung von Vulnerabilität als multidimensionales Konstrukt hilft, die Lebenssituation älterer Menschen differenzierter darzustellen. Vulnerable ältere Erwachsene mit einem kleinen oder unzuverlässigen sozialen Netzwerk und mit Schwierigkeiten beim Zugang zu praktischer Hilfe benötigen zusätzliche externe soziale Unterstützung, um ein hohes Maß an Lebenszufriedenheit zu erreichen.

## Introduction

Overall life satisfaction among older adults is a central indicator in aging research representing a cognitive evaluation process of the subjectively perceived quality of life [7, 23]. Life satisfaction is influenced by numerous factors (typically categorized into health, personal, social, and financial [8]), which are closely linked to social inequality [16]. In this context, poor health, low financial resources or social isolation are considered as typical threats and potential stress factors that may lead to adverse outcomes in life [11]. These factors can unfold an enormous negative impact on the perceived quality of life in old age [2, 21].

The association of risk factors with adverse outcomes—in this case a marked decrease in life satisfaction—comes into full force when the individual cannot draw on protective reserves. The presence of individual risk factors therefore acts as a disposition for vulnerability. The absence of corresponding protective factors subsequently manifests the individual’s vulnerable state. According to Grundy [11, p. 107], “[…] vulnerable older people are defined as those whose reserve capacity falls below the threshold needed to cope successfully with the challenges that they face.” Vulnerability within the biological aging process becomes more and more visible as the physical capacities generally diminish and the individual risk of dying increases [11, 21].

Advantages and disadvantages in old age with regard to health, social relations and financial situation on the individual level are described to emerge within a cumulating process over the life course [6, 9], which leads to increasing heterogeneity in higher age groups [4]. Considering these aspects, a significant dispersion of vulnerability among older adults is expected with varying outcomes [17].

Recent research takes up the idea of heterogeneity in old age and has criticized the empirical implementation of the different dimensions of vulnerability to decreased life satisfaction as merely isolated influencing factors. The simultaneous occurrence of risks in different life domains is usually ignored [17, 22], although inequality research implies that financial, health-related and social disadvantages in old adulthood interact with one another resulting in an accumulation of adverse effects on life satisfaction [2, 26]. Following this argument, Shin et al. successfully assessed multidimensional vulnerability by conducting a latent class analysis with data from the Health and Retirement Study. Along four dimensions of vulnerability (derived from major life domains material, physical, mental, and social), they identified six different vulnerability profiles. Among these, health-related and social vulnerability combined exerts the strongest negative effect on subjective well-being, closely followed by the combination of material, health-related and social vulnerability [22]. These findings support the idea of considering vulnerability to low subjective well-being as a multidimensional phenomenon.

Despite the existence of risk factors that challenge the coping process, the negative impact of stressful events on life satisfaction does not necessarily have to occur if the individual draws on protective reserves that can buffer the negative impact of risk factors on perceived quality of life. Protective factors generally reduce the likelihood of disorders occurring in the presence of stress [11]. They appear either on the personal level (e.g. high self-efficacy [10]) or outside the individual (such as social support [5]). Social support is defined as emotional, material, instrumental or informational help provided by significant members of social networks (i.e. partners, friends, relatives, neighbors) [20]. Referring to vulnerability, it is not sufficient just being a part of a social network. Moreover, the helping exchanges within social networks need to be mobilized in cases of a stressful event [21]. Typically, social support is considered as an external protective factor related to the influence of stressful events on mental health or depressive symptoms in old age (e.g. induced by income inequality) [20]. Although empirical evidence supports the moderating role of this phenomenon, the strength of this buffer effect might be, at some point, overemphasized [20].

Based on the critical interrogations mentioned above, we propose the basic hypothesis that vulnerability acts as a composite measure including risk factors from different dimensions. The aim of this study is twofold: first, we take up the idea of multidimensional vulnerability among older adults and use data from Germany to construct a latent variable reflecting the material, physical, mental and social dimension of vulnerability. Second, we apply this construct in relation to overall life satisfaction. Referring to external coping resources, we expect that the negative direct effect of vulnerability on life satisfaction is moderated by social support.

## Data and methods

For the empirical analyses, the fourth wave of the survey German Health Update (GEDA 2014/2015-EHIS) is used, which was carried out between November 2014 and July 2015. The dataset comprises a sample of 24,016 respondents aged 15 years and older in private households from the German resident population. The core questionnaire of the GEDA survey, based on the third wave of the European Health Interview Survey (EHIS), includes questions on subjective, functional and mental health, chronic diseases, potential causes of diseases, support and social networks, and the use of medical care and treatment. Questions are also asked on other health-related topics, such as care for diabetes mellitus, working conditions and issues related to disease prevention [15, 19]. The relevant sample is limited to persons aged 65 years and older (*N* = 5826).

Multidimensional vulnerability manifests in four dimensions: material, physical, mental and social [22]. These dimensions of vulnerability were measured using the following indicators: income below the poverty risk threshold (60% or less of median monthly income), health-related functional limitations, number of chronic diseases, a lack of close or intimate social relationships, and mental health problems. To measure income below the poverty risk threshold, the two lowest categories of the net monthly equivalent income (≤ 500 €, 500–1000 €) were used as a proxy. The 5‑item scale of activities of daily living (ADL [13, 18]) was used as an indicator of physical health limitations. Individuals who had problems in at least one task of ADL (eating or drinking, getting up from a bed or chair, dressing, using a toilet, etc.) were categorized as having health-related functional limitations. Additionally, the number of chronic conditions and risk factors (asthma, myocardial infarction, stroke, arthritis, diseases of the bowel or the liver, high blood pressure, etc.) were used as an indicator of multimorbidity (three chronic conditions or more). Living alone in the household indicates the social dimension of vulnerability. Mental health problems were assessed by the 8‑item scale of the patient health questionnaire (PHQ) [14] with respondents with a score of five or above indicating mental vulnerability.

Life satisfaction as the outcome variable is measured using an 11-point scale from 0 (“not at all satisfied”) to 10 (“completely satisfied”) [3]. Social support, representing the main effect in this study, is measured using the Oslo 3‑item social support scale [5].

In order to verify that the selected variables can estimate the latent construct of vulnerability on the basis of one dimension, an exploratory factor analysis was carried out in a first step using the principal component factor method. Subsequently, a confirmatory factor analysis (CFA) based on generalized structural equation modeling (GSEM) with probit link functions and including missing values was conducted [24]. To assess the model fit of GSEM in more detail, the root mean square error of approximation (RMSEA) and the comparative fit index (CFI) were calculated as proxies using a linear structural equation model (SEM) with the same variables based on maximum likelihood with missing values (MLMV). As a result of CFA, a continuous latent variable on multidimensional vulnerability was estimated, which was used in the further analyses. In order to investigate the moderation of the relationship between multidimensional vulnerability and life satisfaction by social support, the factor values of the latent variable vulnerability were converted into a manifest variable. From this manifest variable and social support, a multiplicative term was calculated measuring the potential interaction in the subsequent models. These variables were used to estimate linear models on the basis of MLMV in a stepwise procedure. First, the bivariate relationship between life satisfaction and vulnerability was estimated. In the second step, the degree of social support and the interaction term were added to the model. The final model contained further control variables such as gender, age (65–79 years versus 80 years and older) and general self-efficacy. The intercorrelations of the study variables as well as their mean values and standard deviations, are listed in the electronic supplement (Table S1). All statistical analyses were performed using Stata 16.0 [24].

## Results

### Measuring vulnerability

**Fig. 1:**
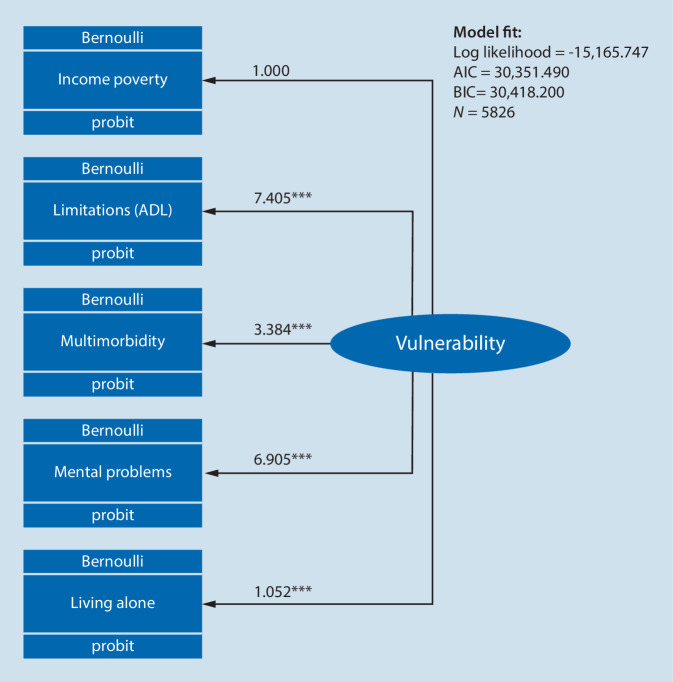
Measurement component of multidimensional vulnerability. Note: unstandardized coefficients, unweighted data, probit link function, missing values included. *AIC* Akaike information criterion, *BIC* Bayesian information criterion, *p < 0.5, **p < 0.01, ***p < 0.001, Bernoulli distribution refers to a discrete random variable which takes the value 1 with probability *p* and the value 0 with probability *q* = 1-*p* (source: GEDA 2014/2015-EHIS, own calculations)

The results of CFA in Fig. 1 show a satisfactory overall fit to the data (log likelihood = −15,165.747, Akaike information criterion (AIC) = 30,351.490, Bayesian information criterion (BIC) = 30,418.200, RMSEA = 0.010, 90% confidence interval, CI [0.000, 0.023], CFI = 0.998) referring to multidimensional vulnerability indicated by material, physical, mental and social vulnerability. As vulnerability is a latent exogenous variable and needs a normalizing constraint, the association with income poverty is constrained to 1; however, within the measurement model, the ADL and multimorbidity scales as well as the PHQ mental health problems scale show the strongest associations with the latent variable vulnerability. The sub-dimensions of physical and mental health thus seem to play the most important role in measuring multidimensional vulnerability among older adults, while living alone shows comparatively lower associations.

**Fig. 2:**
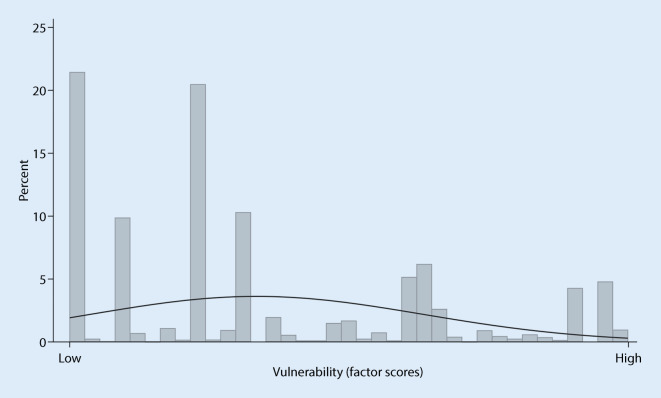
Distribution of vulnerability factor scores in the sample (in %). Note: the solid line shows the approximated normal distribution of the indicator (source: GEDA 2014/2015-EHIS, own calculations)

The latent variable of the measurement model corresponds to a continuous factor score ranging from very low to very high levels of multidimensional vulnerability. About one in five respondents has no vulnerability in any of the dimensions, which corresponds to very low factor scores. Slightly more than half of the respondents have a rather low vulnerability with a prevalent risk in either one or two dimensions. Of the study participants 18% show high to very high vulnerability scores corresponding to the prevalence of risk factors in 3 or more dimensions. Thus, multidimensional vulnerability is characterized by a right-skewed distribution (M < 0.001, SD = 0.151, skewness = 0.707) as shown in Fig. 2. Overall, the factor scores reveal gradual trajectories of vulnerability in much greater detail than would be possible with categorical variables on different groups of vulnerability.

### Multivariate analysis

Vulnerability has a significant negative impact on life satisfaction (β = −0.437; *p* < 0.001; see Model I, Table 1). Adding social support to the regression model (Model II) shows that the effect of vulnerability changes, while social support also significantly influences life satisfaction. These effects persist under control for age, gender and self-efficacy.

**Table 1:** Associations of life satisfaction and vulnerability by social support

Variables	Model I			Model II			Model III		
	β	95% CI		β	95% CI		β	95% CI	
		*LL*	*UL*		*LL*	*UL*		*LL*	*UL*
*Intercept*	4.380***	4.299	4.462	4.081***	3.983	4.180	3.034***	2.888	3.180
*Vulnerability*	−0.437***	−0.457	−0.418	−0.553***	−0.625	−0.481	−0.468***	−0.539	−0.397
*Social support (ref.: poor)*									
Moderate	–	–	–	0.138***	0.107	0.170	0.124***	0.094	0.154
Strong	–	–	–	0.256***	0.225	0.286	0.228***	0.198	0.258
*Age years (ref.: 65–79 years)*									
80+	–	–	–	–	–	–	0.107***	0.084	0.130
*Gender (ref.: male)*									
Female	–	–	–	–	–	–	0.018^n.s.^	−0.004	0.040
Self-efficacy	–	–	–	–	–	–	0.242***	0.217	0.267
*Interaction*									
Vulnerability × Social support	–	–	–	0.153***	0.080	0.226	0.143***	0.072	0.213
*Model fit*									
Log likelihood	–	−8236.497	–	–	−8995.333	–	–	−22,738.257	–
AIC	–	16,478.994	–	–	18,030.667	–	–	45,564.514	–
BIC	–	16,499.004	–	–	18,164.069	–	–	45,857.998	–
CD	–	0.191	–	–	0.231	–	–	0.281	–

*β* standardized coefficient, *CI* confidence interval, *LL* lower limit, *UL* upper limit, *AIC* Akaike information criterion, *BIC* Bayesian information criterion, *CD* coefficient of determination

Models are based on unweighted data (*N* = 5826). n. s.: not significant, *p < 0.5, **p < 0.01, ***p < 0.001

*Source.* GEDA 2014/2015-EHIS, own calculations

The interaction coefficient vulnerability × social support is also found to be significant and points in the opposite direction: The negative effect of vulnerability on life satisfaction is moderated by social support and therefore becomes less pronounced when the level of social support increases. Controlling for age, gender and self-efficacy (Model III), for each unit of social support adds 0.143 to the main effect of vulnerability, reducing the negative coefficient. Accordingly, when social support is strong, the effect of vulnerability on life satisfaction is −0.039 instead of −0.468.

To validate these results, an additional model was calculated without social support and the interaction term, but with a group comparison for the social support categories. This model shows a significant χ^2^-test for group invariance of parameters, which confirms a moderation of the association between vulnerability and life satisfaction by social support (χ^2^(2) = 10.634, *p* = 0.005, data not shown).

The older age group (≥ 80 years) reports higher life satisfaction compared to the younger respondents. Self-efficacy yields a positive coefficient (β = 0.242; *p* < 0.001), while gender does not show a significant correlation with life satisfaction (Table 1).

**Fig. 3:**
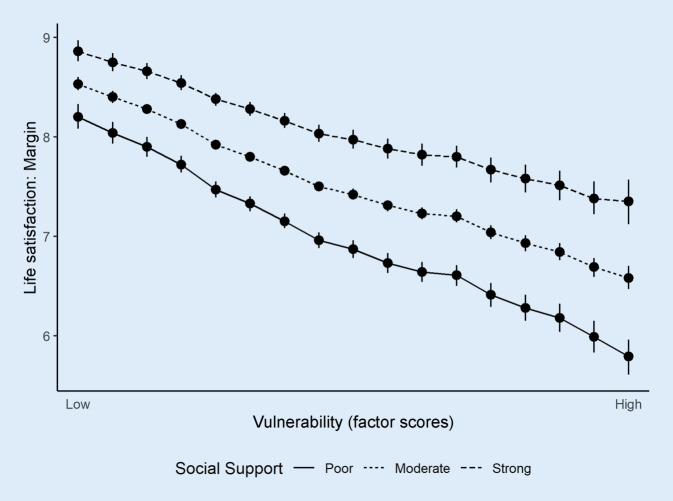
Trajectories of life satisfaction in relation to vulnerability moderated by social support (source. GEDA 2014/2015-EHIS, own calculations). Note: Overall life satisfaction is measured by the following item: ‚Asked in general terms, how satisfied are you with your life overall? 0 = not satisfied at all … 10 = completely satisfied‘.

Figure 3 shows the predicted margins of life satisfaction by level of vulnerability moderated by social support. While there is a general gain in life satisfaction through social support, a stronger effect becomes visible for vulnerable people in particular. The relative difference in life satisfaction increases as the vulnerability level rises, depending on the degree of social support.

## Discussion

The results of this study are twofold: First, by creating a continuous latent variable to represent different degrees of vulnerability, the concept of vulnerability becomes multidimensional and contributes to the current debate over the conceptualization and operationalization of vulnerability [17]. Second, the analyses reveal a negative correlation between this construct and life satisfaction, but that this relationship is mitigated by social support.

Vulnerability theory argues that vulnerability can exist simultaneously in multiple life domains and affect perceived quality of life [25]. The analyses of Shin et al. [22] represent a crucial step in the empirical assessment of multidimensional vulnerability to a marked decrease in perceived quality of life, implying that cumulative life risks affect subjective well-being more than these risks occurring in isolation; however, this approach could lead to conceiving vulnerability as an attributed condition and therefore entails the risk of (unintentional) stigmatization that ends up ascribing a social role to the affected people (“vulnerable elders”), which is linked to certain behavioral expectations of this role (e.g. older people typically as receivers of help). Rather, the present study takes up the idea of the situation of vulnerability [17] and therefore allows adding context to the solely individual vulnerability, represented in the latent vulnerability variable. The situation of vulnerability is described as a set of circumstances in which individuals experience multiple difficulties that may interact to increase the risk of being harmed [17]. This definition makes it obvious that the measurement of vulnerability has to be multi-facetted and differentiated and should focus on a universal concept of vulnerability. Capturing vulnerability as a multidimensional construct and establishing a measurement tool is a promising approach to future research as this could contribute to a vulnerability assessment that becomes comparable across studies and disciplines.

Consistent with previous studies revealing a negative relationship between vulnerability and perceived quality of life on single dimensions [2, 11, 21], the result that multidimensional vulnerability has a negative impact on overall life satisfaction is not surprising. Equally unsurprising in this context is the (robust) observed buffer effect of social support [5, 20]. Less known so far, however, is that with increasing degree of vulnerability the buffering effect of social support also increases, meaning that more vulnerable people profit to a higher degree of social support than less vulnerable people. Regarding the degree of vulnerability, the differences in life satisfaction between poor versus strong social support range from 0.7 to 1.6 points (Fig. 3). At first glance, this might seem quite small but looking at the findings of Guven and Saloumidis [12], a 10% increase on the overall life satisfaction scale is associated with a lower mortality risk of 4 percentage points. Therefore, these differences have substantial significance and social support can thus be regarded as an important protective factor for older adults in vulnerable situations.

These findings have direct practical relevance, as they show that older adults facing multidimensional vulnerability particularly need special external support to achieve a higher level of life satisfaction. Identifying the multidimensionally vulnerable is a major task for actors in community work (e.g. social agencies or service providers) and could benefit from a standardized and generally accepted assessment and screening of vulnerability. Moreover, potential services of external social support are at least as broad as the composition of vulnerability in older adults. To create helpful measures, the federal and local governments should collaborate with scientific research on models of external interventions based on subsidiarity and solidarity. This call for a mix of tailored interventions addresses actors on different levels, such as public health (regarding physical and mental well-being), the welfare state (regarding financial provision) and the community (regarding support in everyday tasks).

In the light of this multidimensional perspective, aging describes a process that involves the accumulation of successive and time-variant changes in different domains within different settings. Essentially, this involves intra-individual and inter-individual differences in the process of development and adaptation and is based on life span psychology [1]. The process of change does not affect all the domains of a particular individual at the same time and to the same extent, which leads to the simultaneous existence of more vulnerable and more resistant domains. This results in a highly individual combination of domain-specific expressions that are unique in each human being. Unravelling this accumulation and making it possible to measure it in its complexity is a promising research goal, as this is relevant for policy makers and practitioners, but above all, for the individuals themselves because it reveals their needs and strengths and enables tailored interventions based on individual profiles.

## Limitations

Increasing the significance of the study results would entail the following changes in the study design:

- Use of longitudinal data in order to (1) identify a baseline regarding overall life satisfaction and (2) investigate for cause and effect.
- Oversampling older persons with multiple risk factors to gain a deeper understanding and a more differentiated picture of vulnerability in old age.

## Practical recommendations

Our findings can be useful in suggesting approaches for practitioners:

- The focus of practical interventions should be on the individual situation of vulnerability, and thus a “one size fits all” approach should be avoided and replaced by tailored interventions.
- Interventions to promote social support and life satisfaction appear to be most effective among the most vulnerable. It follows that identifying individuals with high levels of vulnerability is essential and directing them to appropriate interventions promises the most positive outcomes.
- In order to increase life satisfaction of older vulnerable people living alone, social relations in the local environment (e.g. neighbors) should be strengthened.
- Targeted urban and social planning can promote support for vulnerable older people in their immediate social environment by enabling close links between individuals and their context.
- Developing measures to improve perceived life satisfaction can be more effective if older adults confronted with multidimensional vulnerability are already involved early in the planning process.

### Supplementary Information

Tab. S1: Correlations, means, standard deviations, minimum and maximum of the study variables

## Declarations

For this article no studies with human participants or animals were performed by any of the authors. All studies mentioned were in accordance with the ethical standards indicated in each case.
