# Supplementary material for: Multidimensional vulnerability among older adults in Germany: Social support buffers the negative association with life satisfaction
Source: Z Gerontol Geriatr. 2022 Dec 2;56(8):654–60. doi: 10.1007/s00391-022-02142-3 (PMC10709245; doi:10.1007/s00391-022-02142-3)
Supplement: Supplementary file 1 — Tab. S1: Correlations, means, standard deviations, minimum and maximum of the study variables [file 391_2022_2142_MOESM1_ESM.docx]

Supplement 1

Table S1: Correlations, means, standard deviations, minimum and maximum of the study variables

| Variable | 1 | 2 | 3 | 4 | 5 | M | SD | Min | Max |
| --- | --- | --- | --- | --- | --- | --- | --- | --- | --- |
| 1. Life satisfaction | - |  |  |  |  | 7.89 | 1.80 | 0 | 10 |
| 2. Vulnerability | -0.44^***^ | - |  |  |  | <0.00 | 0.15 | -0.17 | 0.34 |
| 3. Social support^a^ | 0.26^***^ | -0.15^***^ | - |  |  | 2.08 | 0.67 | 1 | 3 |
| 4. Gender^b^ | -0.04 | 0.12^***^ | 0.02 | - |  | 0.50 | 0.50 | 0 | 1 |
| 5. Age^c^ | -0.05^**^ | 0.26^***^ | -0.04^*^ | 0.01 | - | 0.19 | 0.39 | 0 | 1 |
| 6. Self-efficacy | 0.39^***^ | -0.45^***^ | 0.15^***^ | -0.08^***^ | -0.27^***^ | 4.00 | 0.39 | 1 | 5 |
| ^a^ 1 low, 2 moderate, 3 high; ^b^ 0 male, 1 female; ^c^ 0 young-old (65-79 years), 1 old-old (80+). | | | | | | |  |  |  |
| N = 5,597-5,826; *** p < .001; ** p < .01; * p < .05. | | |  |  |  |  |  |  |  |
